# Effect of carotenoid class and dose on the larval growth and development of the critically endangered southern corroboree frog

**DOI:** 10.1093/conphys/coz009

**Published:** 2019-03-13

**Authors:** Emma P McInerney, Aimee J Silla, Phillip G Byrne

**Affiliations:** School of Earth, Atmospheric and Life Sciences, University of Wollongong, NSW, Australia

**Keywords:** Amphibian, antioxidant, captive breeding, carotenoid, larvae, nutrition

## Abstract

Dietary carotenoids are expected to improve vertebrate growth and development, though evidence for beneficial effects remains limited. One reason for this might be that few studies have directly compared the effects of carotenoids from different classes (carotenes versus xanthophylls) at more than one dose. Here, we tested the effect of two doses of dietary β-carotene and lutein (representing two different carotenoid classes) on the growth and development of larval southern corroboree frogs (*Pseudophryne corroboree*). Individuals were supplemented with either β-carotene or lutein at one of two doses (0.1 mg g^−1^, 1 mg g^−1^), or given a diet without carotenoids (control). Each dietary treatment included 36 replicate individuals, and individuals remained on the same diet until metamorphosis (25–39 weeks). We measured larval survival, larval growth (body length), time to metamorphosis, metamorphic body size (mass and SVL), and body condition. Lutein had no detectable effect on larval growth and development. However, larvae receiving a high dose (1 mg g^−1^) of β-carotene metamorphosed significantly faster than all other dietary treatments, despite no significant differences in growth rate. This result indicates that β-carotene supplementation in *P. corroboree* has positive effects on development independent of growth effects. Our study provides new evidence for differential effects of carotenoid class and dose on vertebrate development. From a conservation perspective, our findings are expected to assist with the recovery of *P. corroboree* by expediting the generation of frogs required for the maintenance of captive insurance colonies, or the provision of frogs for release. More broadly, our study highlights the potential for dietary manipulation to assist with the *ex situ* management of threatened amphibian species worldwide.

## Teaser

This study shows that dietary β-carotene reduces time to metamorphosis in a critically endangered frog, independent of growth effects. Supplementing captive frogs with dietary β-carotene could assist with amphibian conservation breeding programs globally.

## Introduction

The establishment of conservation breeding programs is a primary conservation action for threatened animal species worldwide (Gascon, 2007; Conde *et al.*, 2013; Tapley *et al.*, 2015). Conservation breeding programs aim to safeguard threatened species *ex situ*, while also providing individuals for release *in situ* (Conde *et al.*, 2013). These programs are widely considered to be critical for insuring species survival, but many programs experience low success due to difficulties associated with efficiently producing large numbers of viable individuals (Dolman *et al.*, 2015). In many cases, this impediment relates to a lack of knowledge regarding nutritional requirements (Pough, 2007; Thangaraj and Lipton, 2008; Ferrie *et al.*, 2014). Accordingly, there has been an emerging focus on manipulating the diets of captive animals, with a view toward improving the growth and development of individuals, as well as the size and overall viability of captive populations (Thangaraj and Lipton, 2008; Li *et al.*, 2009; Ogilvy *et al.*, 2012; Byrne and Silla, 2017).

One group of micronutrients expected to improve the growth and development of captive animals are carotenoids. Carotenoids are a group of over 600 hydrocarbon compounds that animals cannot synthesize *de novo* and must gain through their diet (Svensson and Wong, 2011). Dietary carotenoids are expected to improve growth and development in several ways. First, carotenoids are powerful antioxidants with the capacity to quench and stabilize reactive oxygen species (ROS) generated during periods of high metabolic activity (Loft *et al.*, 1994), such as stages of rapid growth and development (Stoks *et al.*, 2006; Metcalfe and Alonso‐Alvarez, 2010). By binding to ROS, carotenoids limit damage caused to cells, tissue, and DNA, and may promote rapid cell division and differentiation (Miller *et al.*, 1996; Stahl and Sies, 2003, 2005). Second, the availability of dietary carotenoids is expected to improve the general nutritional quality of the diet allowing more resources (such as lipids and proteins) to be allocated to bodily processes such as immune function, somatic growth, organ development, and limb development (Ogilvy and Preziosi, 2012). Third, provitamin A carotenoids (α-carotene, β-carotene, and β-cryptoxanthin) are precursors to the formation of retinoic acid (Balmer and Blomhoff, 2002, 2006), which has been shown to influence gene expression and the production of proteins that have direct impacts on tissue and organ development (Balmer and Blomhoff, 2002, 2006).

To date, a number of dietary manipulation studies have investigated the influence of carotenoid supplementation on growth and development in various vertebrate groups (Cucco *et al.*, 2006; Wang *et al.*, 2006; Ogilvy *et al.*, 2012; McCartney *et al.*, 2014; Pham *et al.*, 2014; Cothran *et al.*, 2015; Byrne and Silla, 2017). Despite this research, evidence for positive effects remains equivocal. The effect of dietary carotenoids on growth and development is likely to be dependent on two main factors; the class of carotenoid tested, and the dose at which the carotenoid supplement is administered. Carotenoids split into two classes: (1) the carotenes (e.g. α-carotene, β-carotene, lycopene) which contain only hydrogen and carbon atoms and (2) the xanthophylls (e.g. zeaxanthin, lutein, astaxanthin) which contain at least one oxygen atom (Miller *et al.*, 1996; Stahl and Sies, 2003, 2005; Pérez-Rodríguez, 2009; Nagao, 2011). In principle, the unique structure of carotenoids in each class will determine how they influence vertebrate growth and development (Stahl and Sies, 2005; Pérez-Rodríguez, 2009). For instance, carotenes are known to be important for the development of tissue, organs, and the central nervous system (Blomhoff and Blomhoff, 2006), and can increase immune activity (Fitze *et al.*, 2007). In contrast, xanthophylls can act as anti-inflammatories, and may improve immunity and general health, potentially supporting rapid growth (Koutsos *et al.*, 2006). It has also recently been shown that the xanthophyll lutein plays an essential role in bone mineralization and development (Takeda *et al.*, 2017). Due to fundamental differences in the structure and function of carotenes and xanthophylls, there is reason to expect that carotenoids from these classes will differ in their capacity to support growth and development. Despite this, most past studies have only tested the effects of carotenoids from a single class (most commonly β-carotene, lutein, or zeaxanthin) (Cucco *et al.*, 2006; Larcombe *et al.*, 2010; Butler and McGraw, 2012; Orledge *et al.*, 2012; Sutherland *et al.*, 2012; McCartney *et al.*, 2014; Cothran *et al.*, 2015). Studies directly comparing the effects of carotenes and xanthophylls are urgently needed to deepen our understanding of how different carotenoid classes influence vertebrate growth and development.

In addition to the importance of carotenoid class, the potential for dose effects has also been largely overlooked. Based on optimization theory, it is predicted that there will be an optimal dose at which carotenoids should be administered (Cucco *et al.*, 2006; Cothran *et al.*, 2015). Supraoptimal doses can potentially be harmful to individuals because excessive carotenoid supplementation can cause a pro-oxidative effect that damages proteins, lipids, and DNA (Palozza, 1998; Catoni *et al.*, 2008). By contrast, carotenoid doses that are too low may have limited impact or no detectable effect on growth and development. Critically, optimal doses are also expected to vary substantially among species (Pérez-Rodríguez, 2009; Koch *et al.*, 2016). Fundamental physiological differences between species are likely to affect their carotenoid requirements (Pérez-Rodríguez, 2009; Koch *et al.*, 2016). For example, it is expected that species with energetically costly carotenoid-based ornamentation will have higher carotenoid requirements and a greater amount of circulating carotenoids than dull species (Pérez-Rodríguez, 2009; Koch *et al.*, 2016). Furthermore, differences in species’ life histories are likely to influence their access to carotenoids and ability to utilize them (Tella *et al.*, 2004, Koch *et al.*, 2016). While past studies generally offer little justification for the carotenoid dose tested (see Koch *et al.*, 2016), interspecific variation in responses to carotenoid supplementation emphasizes the need for a better understanding of carotenoid dose thresholds between taxa.

Amphibians are an important taxon to investigate the effects of carotenoid class and dose on growth and development because approximately 40% of known amphibian species are threatened with extinction (IUCN, 2018), and many are the subject of conservation breeding programs (Tapley *et al.*, 2015; Harding *et al.*, 2016). Understanding how nutritional conditions experienced in captivity affect growth and development is expected to significantly improve our capacity to produce viable individuals, which stands to improve our ability to maintain viable colonies and generate animals for release. Previous studies have highlighted the potential for dietary carotenoid supplementation to improve amphibian growth and development, particularly during the larval growth period, and at metamorphic climax when ROS production is high (Inoue *et al.*, 2004; Ogilvy and Preziosi, 2012; Ogilvy *et al.*, 2012; Szuroczki *et al.*, 2016). Despite these advances, few past studies have directly compared carotenoids from different classes and very few have tested multiple doses in amphibians (but see Ogilvy *et al.*, 2012; Cothran *et al.*, 2015; Keogh *et al.*, 2018). The aim of this study was to investigate the influence of two dietary carotenoids (β-carotene and lutein), each supplemented at two doses (0.1 mg g^−1^ and 1 mg g^−1^), on the growth and development of the critically endangered southern corroboree frog, *Pseudophryne corroboree*. Dietary carotenoids have previously been found to affect *P. corroboree* escape-response, skin colouration, and cutaneous bacterial communities (Silla *et al.*, 2016; Umbers *et al.*, 2016; Edwards *et al.*, 2017; McInerney *et al.*, 2017). Based on this knowledge, *P. corroboree* is expected to require dietary carotenoids for multiple physiological processes. As β-carotene and lutein are from different carotenoid classes (β-carotene is a carotene, lutein is a xanthophyll), we predicted that these carotenoids would affect growth and development differently. In addition, we predicted that a higher carotenoid dose (approximating doses previously found to have positive effects on fitness-determining traits in *P. corroboree* and other frog species) would expedite growth and development.

## Methods

All procedures described in this study were conducted following evaluation and approval of the University of Wollongong’s Animal Ethics Committee (approved number AE 16/19). All applicable institutional/or national guidelines for the care and use of animals were followed.

### Study species

**Figure 1:**
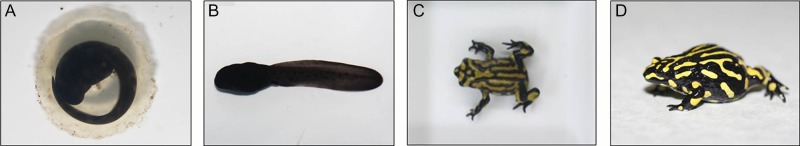
The life-stages of *P. corroboree.* Shown in the (**A**) embryonic life-stage (development within the jelly capsule), (**B**) larval life-stage (tadpole post-hatching), (**C**) juvenile life-stage (newly metamorphosed frog) and (**D**) adult life-stage. Photographs A, B, and D are courtesy of A.J. Silla, photograph C is courtesy of E.P. McInerney.

*Pseudophryne corroboree* is a small (25–30 mm, adult snout-vent length) Myobatrachid anuran endemic to the Snowy Mountain region of New South Wales (Osborne, 1991). It is classified as critically endangered and is the subject of a large-scale conservation breeding program (Hunter *et al.*, 2007; IUCN, 2018). *P. corroboree* breeds over summer (January and February), and eggs are laid in a terrestrial nest (Osborne, 1991). The eggs remain in diapause until heavy autumn rainfall floods nest sites, and hypoxic conditions trigger hatching. *P. corroboree* larvae (tadpoles) develop over winter in small, vegetated pools and reach metamorphic climax in early summer (Osborne, 1991). The life stages of *P. corroboree* are shown in Fig. 1. The diet of *P. corroboree* larvae is known to consist of algae (Osborne, 1991), which is rich in carotenoids (Lichtenthaler, 1987).

### Husbandry and nutrition

A total of 180 fertilized *P. corroboree* eggs from nine clutches were obtained from a captive bred colony held at Taronga Zoo, Australia. Eggs were laid between February and March 2017 and were maintained at Taronga Zoo until they were in a late stage of development (Gosner stage 26; Gosner, 1960) and ready to hatch. Eggs were transported to the Ecological Research Centre at the University of Wollongong on 1 May 2017. Following arrival, clutches were separated into plastic containers (31.9 cm × 17.8 cm × 20 cm) and stimulated to hatch via flooding with 500 ml of reverse-osmosis (R.O.) water. All larvae hatched within 5 days of flooding, and within 24 hrs of hatching were transferred to individual experimental containers (10.5 cm × 10 cm × 10 cm) filled with 550 ml of R.O. water. Three times a week, experimental containers received a 50% water change using an automated irrigation system (PIS Irrigation Systems, Australia) connected to an R.O. water system (Sartorius Stedim Biotech, Germany) to expel excess food and excrement. Any additional food and excrement was siphoned out weekly from each experimental container using a 30-ml plastic syringe connected to a 15 cm length of aquarium tubing (3 mm ID). Individuals were kept in an artificially illuminated, constant temperature room maintained at 12°C with a 11.5:12.5-h light: dark cycle, including a 15-min twilight period at both dawn and dusk. Individuals were also given 1-hr of UV-B light per day provided by fluorescent strip bulbs (Reptisun 10.0 36” bulb; Pet Pacific, Australia) suspended approximately 20 cm above each container.

Following hatching, larvae were assigned to one of five dietary treatments (see Supplementary Table S1). Briefly these diets were; (1) control (0 mg g^−1^), (2) low-dose β-carotene (0.1 mg g^−1^), (3) high-dose β-carotene (1 mg g^−1^), (4) low-dose lutein (0.1 mg g^−1^) and, (5) high-dose lutein (1 mg g^−1^) (see Supplementary Table S1). Total carotenoids in the control diet were <0.015 mg g^−1^ (see Supplementary Table S2). Carotenoid doses were selected as they are within the range previously used to investigate the effects of dietary carotenoid supplementation on amphibian growth and development, with positive results (Ogilvy *et al.*, 2012, Keogh *et al.*, 2018). Cellulose microcrystalline powder (435 236; Sigma-Aldrich, Castle Hill, NSW) was added to experimental diets to balance feed quantity (see Supplementary Table S1). Previous studies have used cellulose as a dietary bulking agent because it is not considered to have any significant nutritional value, is tasteless and odorless (Dias *et al.*, 1998; Keogh *et al.*, 2018). Each dietary treatment mix was prepared by suspending 2 g of fish flake (75:25 Sera Flora/Sera Sans: SERA, Germany) supplemented with 2 mg of the corresponding carotenoid/cellulose mix in 20 ml of R.O. water. Feed was stored in 10 ml plastic syringes, frozen, and then thawed immediately prior to feeding. Larvae received two homogenized droplets of food three times a week (dry mass = 0.0074–0.0099 g) for the first eight weeks of the experimental period. To accommodate increased metabolic demands, after this time food quantity was increased to four droplets (dry mass = 0.0151–0.0175 g) three times a week until the beginning of metamorphic climax (forelimb emergence; Gosner stage 42).

At the beginning of metamorphic climax, individuals were moved from their larval experimental containers into metamorphic containers (18 cm × 10 cm × 12cm). Metamorphic containers were filled with 100 ml of R.O. water and a graduated layer of aquarium pebbles (approximately 0.5 cm thick at low point, 4 cm thick at high point; Tuscan Path, Australia) to allow individuals to crawl from the water. As larvae do not feed during metamorphic climax they were not provided with food. When individuals crawled out of the water they were transferred to sub-adult containers (10.5cm × 10cm × 10cm) which consisted of a layer of aquarium pebbles (approximately 2cm deep; Tuscan Path, Australia), covered by sphagnum moss (*Sphagnum cristatum*) (approximately 5cm deep; Brunnings, Australia). Containers were misted with R.O. water twice a week (Wednesday and Sunday) to keep the substrate moist.

### Quantifying larval survival and growth

To quantify larval survival, individuals were checked every two days and given a score of either alive or dead. To quantify larval growth, each individual was photographed fortnightly from the commencement of dietary treatments (week 0; 8 May 2017) until the first larva reached metamorphic climax (week 20; 29 September 2017). Photographs were taken using a Canon EOS 1000D camera positioned 40 cm above larval experimental containers and attached to a copy stand for image stability. Images were later used to quantify body length (the sum of head and tail lengths) for each individual using image analysis software (ImageJ; Schneider *et al.*, 2012). Each measure was calculated twice and averaged.

### Quantifying metamorphic body mass, snout-vent length, body condition and time to metamorphosis

Time to metamorphosis was recorded as time to full tail absorption (Gosner stage 46) from the start of the experimental period. On the day of full tail absorption, individuals were blotted with an absorbent tissue, weighed, and photographed next to a scale. Images were later used to quantify snout-vent length (SVL) using image analysis software (ImageJ; Schneider *et al.*, 2012). SVL was measured twice and averaged. Body condition was then calculated by taking the residuals of a regression analysis of body mass (g) against SVL (mm).

### Statistical analysis

To test the effect of diet treatment on larval survival, we used a likelihood ratio Chi-squared test. Within each statistical model dietary treatment was the explanatory variable and survival to metamorphosis was the response variable. To test the effect of dietary treatment on larval growth we ran 11 separate linear mixed effects models (LME) with restricted maximum likelihood for experimental weeks 0, 2, 4, 6, 8, 10, 12, 14, 16, 18 and 20. To avoid statistical issues with incomplete data, only individuals surviving to metamorphosis were included in these models (Control treatment; *n* = 31, Low-dose β-carotene; *n* = 29, High-dose β-carotene; *n* = 29, Low-dose lutein; *n* = 26, High-dose lutein; *n* = 26). Within each model, dietary treatment was the fixed factor, clutch (1–9) was included as a random factor, and body length was the response variable. Prior to analysis all data for LME models were log transformed to meet the assumptions of normality and homogeneity. Where a clutch effect was found, post-hoc two-way analysis of variance (ANOVA) tests were conducted for each experimental week to determine if there was a significant dietary treatment by clutch interaction. We tested the effects of dietary treatment on metamorphic body mass, SVL, body condition, and time to metamorphosis at two separate metamorphic points of each dietary treatment. The first metamorphic point was when 50% of individuals from each dietary treatment had metamorphosed (Control treatment; *n* = 15, Low-dose β-carotene; *n* = 14, High-dose β-carotene; *n* = 14, Low-dose lutein; *n* = 13, High-dose lutein; *n* = 13) and the second, was when 100% of individuals had metamorphosed (Control treatment; *n* = 31, Low-dose β-carotene; *n* = 29, High-dose β-carotene; *n* = 29, Low-dose lutein; *n* = 26, High-dose lutein; *n* = 26). To test for the effect of dietary treatment at both metamorphic points we ran eight LME models where dietary treatment was the fixed factor and clutch (1–9) was included as a random factor. Response variables were either; (i) metamorphic body mass, (ii) metamorphic SVL, (iii) metamorphic body condition, or (iv) time to metamorphosis (days). Body mass, SVL, and body condition all met the assumptions of normality and homogeneity. However, time to metamorphosis data were log transformed to improve normality and variances. Where significant effects were found between dietary treatments, post-hoc Tukey’s HSD tests for multiple comparisons were used. All statistical analyses were performed using JMP Pro 13 (SAS Institute Inc. North Carolina, USE).

## Results

### Effect of dietary treatment on larval survival

Overall, 78.9% (142/180) of larvae survived through to metamorphosis. Larval survival was higher in the control treatment (86.1%) than in the carotenoid-supplemented treatments (Low-dose β-carotene: 80.6%, High-dose β-carotene: 83.3%; Low-dose lutein; 72.2%; High-dose lutein; 72.2%), but this difference was not significant (Chi-squared: *x*^2^ = 3.536, *df* = 4, *P* = 0.472).

### Effect of dietary treatment on larval growth

**Figure 2:**
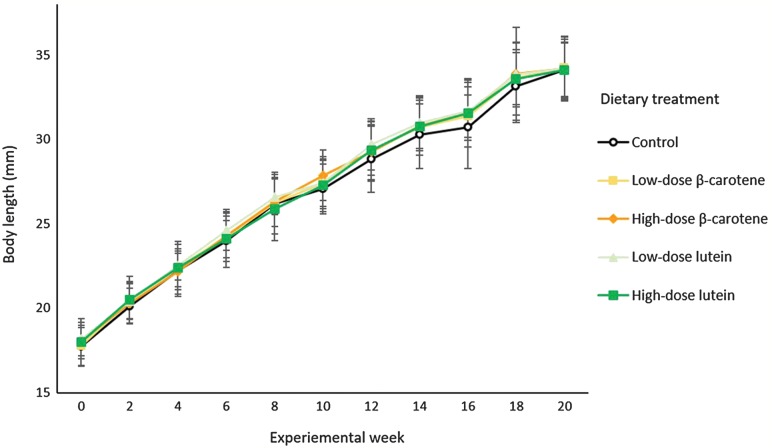
Effect of dietary treatment over the 20-week experimental period on larval body length (mm) of *P. corroboree*. Data are untransformed means ± SD (*n =* 26–31 individuals per treatment).

The body length of larvae increased linearly over the experimental period (*n* = 20 weeks), with individuals reaching an average (±SD) body length of 34.158 ± 1.744 mm by the end of the 20 weeks (Fig. 2). There was no significant effect of dietary treatment on larval body length at any of the sampling weeks (LME weeks 0– 20: *P* > 0.05; Table 1). There was an effect of clutch on body length in week 20 (ANOVA: *F*_*8,8*_ = 2.641, *P* = 0.012), but there was no significant clutch by dietary treatment interaction (ANOVA: *F*_*32,32*_ = 0.597, *P* = 0.951).

**Table 1.** The effect of dietary treatment on the body length (mm) of larval *P. corroboree* over the 20-week experimental period. Data shown are means ± SD, and statistics presented are the result of 11 separate LME models (*n* = 26–31 individuals per treatment).

Experimental week	Mean body length ± SD (mm)					*F*_df_	*P*
	Control (*n* = 31)	Low-dose β-carotene (0.1 mg g^–1^) (*n* = 29)	High-dose β-carotene (1 mg g^–1^) (*n* = 29)	Low-dose lutein (0.1 mg g^–1^) (*n* = 26)	High-dose lutein (1 mg g^–1^) (*n* = 26)		
Week 0	17.734 ± 1.205	17.775 ± 1.196	18.081 ± 1.065	18.183 ± 1.000	17.986 ± 1.387	0.804_4, 128.9_	0.525
Week 2	20.128 ± 1.052	20.462 ± 1.103	20.295 ± 1.172	20.423 ± 1.063	20.492 ± 1.398	0.551_4, 128.8_	0.699
Week 4	22.233 ± 1.541	22.403 ± 1.125	22.163 ± 1.079	22.518 ± 0.869	22.400 ± 1.558	0.337_4, 129.3_	0.853
Week 6	23.975 ± 1.209	24.240 ± 1.229	24.296 ± 1.327	24.575 ± 1.163	24.129 ± 1.720	0.726_4, 129.3_	0.576
Week 8	26.170 ± 1.758	26.206 ± 1.829	26.305 ± 1.450	26.593 ± 1.051	25.886 ± 1.871	0.708_4, 130.3_	0.588
Week 10	27.058 ± 1.471	27.413 ± 1.538	27.867 ± 1.499	27.395 ± 1.365	27.264 ± 1.558	1.241_4, 129.6_	0.297
Week 12	28.816 ± 1.971	29.363 ± 1.505	29.279 ± 1.777	29.695 ± 1.531	29.352 ± 1.734	1.047_4, 129.7_	0.386
Week 14	30.283 ± 2.022	30.713 ± 1.370	30.813 ± 1.758	30.972 ± 1.532	30.765 ± 1.686	0.721_4, 129.7_	0.579
Week 16	30.690 ± 2.435	31.389 ± 1.251	31.565 ± 2.011	31.662 ± 1.706	31.539 ± 1.979	1.305_4, 130.9_	0.272
Week 18	33.150 ± 2.162	33.607 ± 1.538	33.874 ± 2.750	33.823 ± 1.903	33.585 ± 2.162	0.477_4, 129.7_	0.753
Week 20	34.085 ± 1.662	34.234 ± 1.702	34.193 ± 1.882	34.184 ± 1.933	34.096 ± 1.625	0.020_4, 129.3_	0.999

### Effect of dietary treatment on metamorphic body mass, snout-vent length and body condition

**Figure 3:**
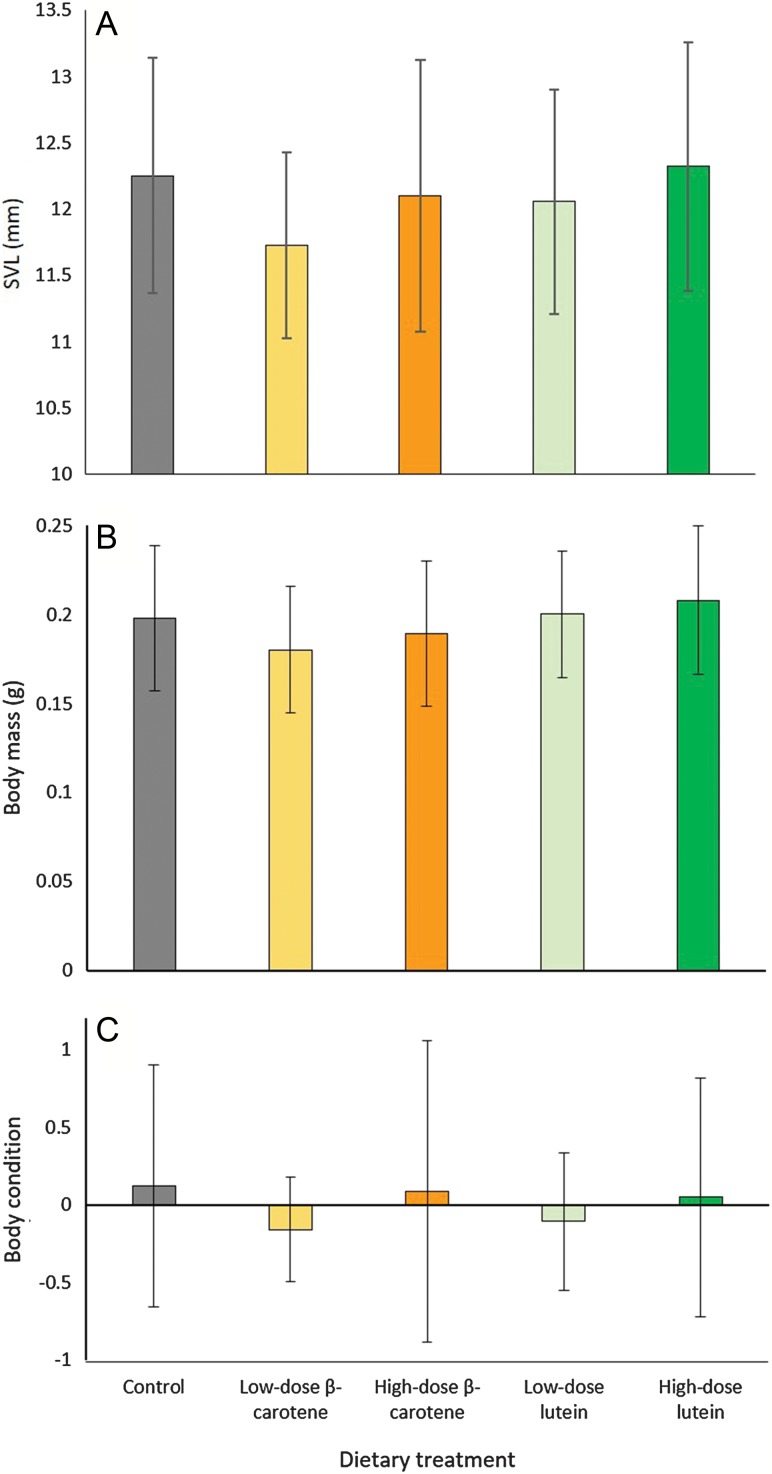
Effect of dietary treatment on; (**A**) SVL for 50% of individuals to metamorphose from each dietary treatment, (**B**) mass for 50% of individuals to metamorphose from each dietary treatment, (**C**) body condition for 50% of individuals to metamorphose from each dietary treatment. Data are untransformed means ± SD (*n* = 13–15 individuals per treatment).

**Figure 4:**
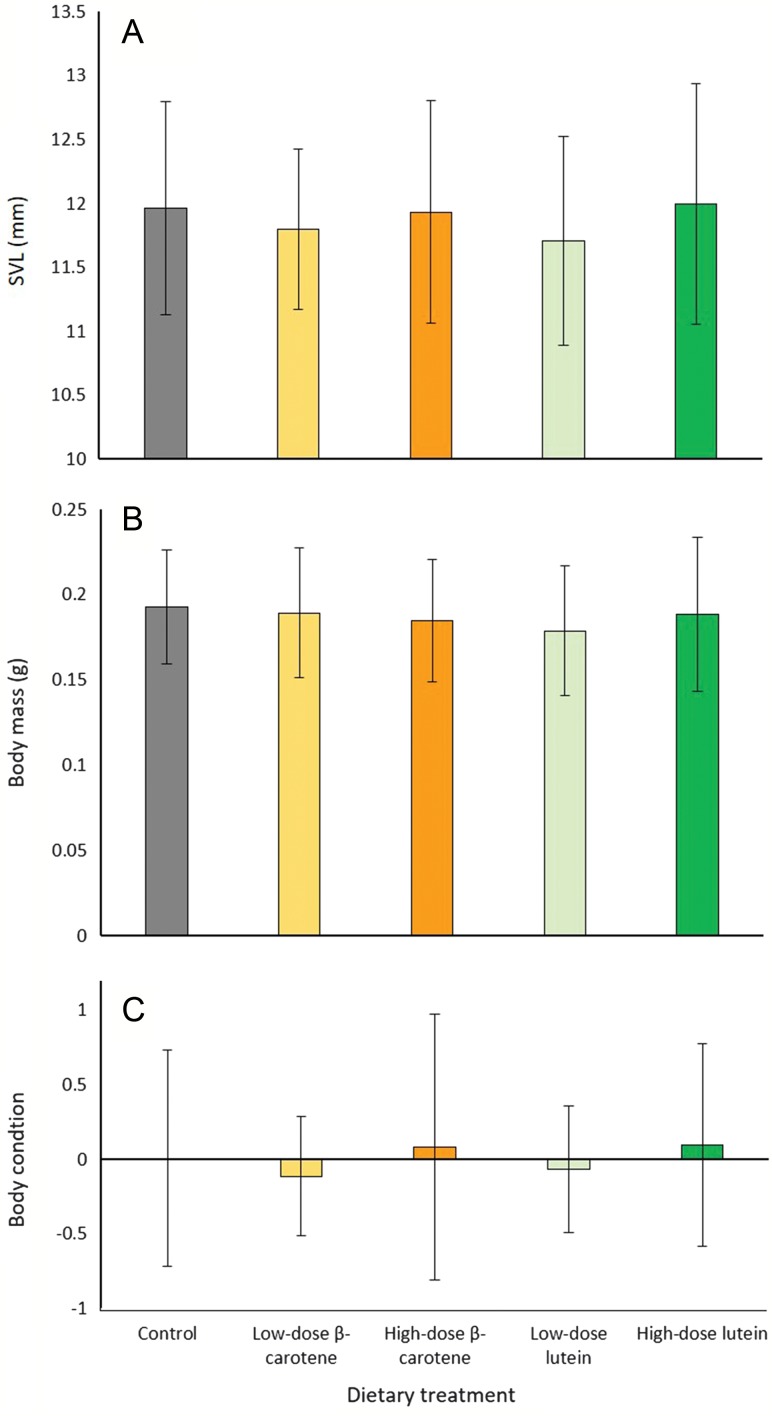
Effect of dietary treatment on; (**A**) SVL for 100% of individuals to metamorphose from each dietary treatment, (**B**) mass for 100% of individuals to metamorphose from each dietary treatment and (**C**) body condition for 100% of individuals to metamorphose from each dietary treatment. Data are untransformed means ± SD (*n* = 26–31 individuals per treatment).

The average (±SD) body mass of individuals at the time of metamorphosis was 0.187 ± 0.038 g (range = 0.096 – 0.307 g) and the average (±SD) SVL was 11.881 ± 0.820 mm (range = 9.786 – 14.199 mm). For the first 50% of individuals to metamorphose from each treatment, there was no significant effect of dietary treatment on SVL (LME: *F*_*4,58.28*_ = 0.937, *P* = 0.449) (Fig. 3A), body mass (LME: *F*_*4,59.27*_ = 0.958, *P* = 0.437) (Fig. 3B), or body condition (LME: *F*_*4,60.58*_ = 0.405, *P* = 0.805) (Fig. 3C). Similarly, for all individuals to metamorphose, there was no effect of dietary treatment on SVL (LME: *F*_*4,130*_ = 0.602, *P* = 0.662) (Fig. 4A), body mass (LME: *F*_*4,129.1*_ = 0.596, *P* = 0.666) (Fig. 4B), or body condition (LME: *F*_*4,129.9*_ = 0.517, *P* = 0.724) (Fig. 4C).

### Effect of dietary treatment on the time to metamorphosis

**Figure 5:**
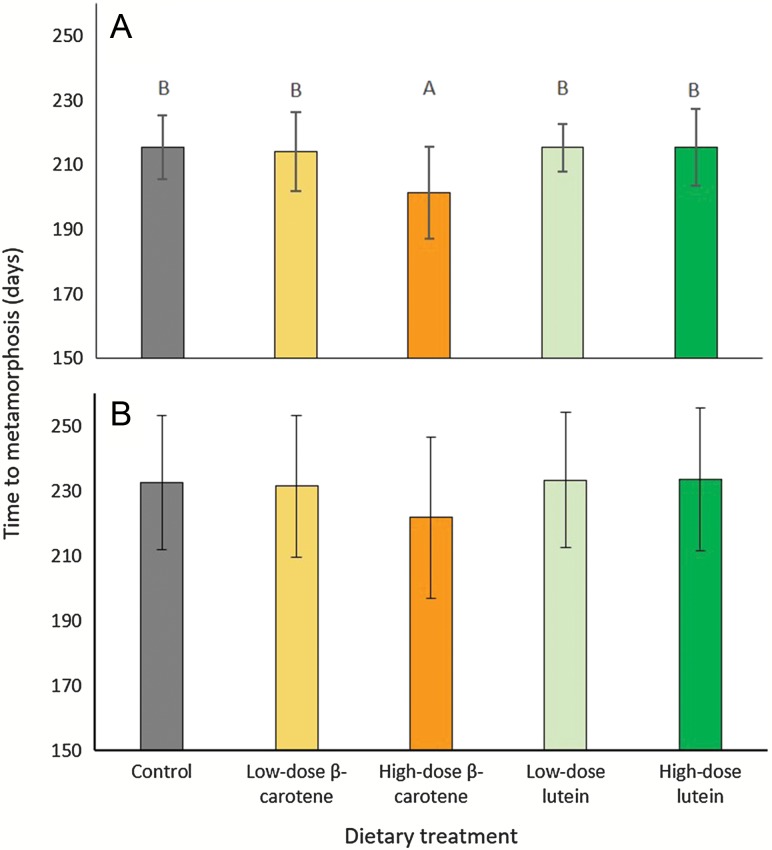
Effect of dietary treatment on the time to metamorphosis (days) of *P. corroboree* for (**A**) 50% of individuals to metamorphose from each dietary treatment (*n* = 13–15 individuals per treatment), and (**B**) 100% of individuals to metamorphose from each dietary treatment (*n* = 26–31 individuals per treatment). Data are untransformed means ± SD. Treatments that share a letter are not significantly different from one another.

Overall, individuals took an average (±SD) of 230.560 ± 22.207 days to metamorphose (range = 178 – 270 days). For the first 50% of individuals to metamorphose, there was a significant effect of dietary treatment on time to metamorphosis (LME: *F*_*4,59.81*_ = 4.111, *P* = 0.005). On average, individuals from the high-dose β-carotene dietary treatment metamorphosed 14–15 days faster than individuals from all other dietary treatments (Fig. 5A). Overall, once all individuals had metamorphosed, on average individuals from the high-dose β-carotene dietary treatment still metamorphosed faster than individuals from all other diet treatments (Fig. 5B), though this was not statistically significant (LME: *F*_*4,129.3*_ = 1.587, *P* = 0.182).

## Discussion

Carotenoid class and dose are two factors likely to influence whether dietary carotenoids have a significant effect on vertebrate growth and development. However, few studies have directly compared the effects of carotenoids from different classes at more than one dose, which may explain the equivocal nature of past findings. The present study tested the effect of dietary carotenoids on the growth and development of the southern corroboree frog, *Pseudophryne corroboree.* Two dietary carotenoids (β-carotene and lutein) representing two carotenoid classes (carotenes and xanthophylls, respectively) were administered at two doses: low (0.1 mg g^−1^) and high (1 mg g^−1^). While dietary treatment had no significant effect on larval growth, metamorphic body size (body mass and SVL), or metamorphic body condition, individuals from the high-dose β-carotene treatment metamorphosed significantly faster than those from all other dietary treatments (when considering the first 50% of individuals to metamorphose). This result indicates that high-dose β-carotene supplementation expedited development, independent of growth effects.

Across all dietary treatments, larvae grew at a similar linear rate and reached metamorphosis at approximately the same body size and body condition. This finding is consistent with the findings of a previous study in *P. corroboree* which reported no effect of a broad-spectrum carotenoid supplement (containing β-carotene, lutein, and various other carotenoids in combination) on either larval growth rate or body size at metamorphosis (Byrne and Silla, 2017). Dietary carotenoids might not improve growth rate in *P. corroboree* because this species has a protracted larval phase and a very slow growth rate. In nature, *P. corroboree* larvae hatch in austral autumn, then develop in cold winter ponds and do not metamorphose until summer (Colefax, 1956; Osborne, 1991). In our study, rearing temperature remained at 12°C (to simulate average temperatures experienced by larvae in nature), and individuals took on average 7.5 months to metamorphose. Slow growth rate in ectothermic vertebrates is expected to reduce metabolic activity and lower oxygen consumption (Stoks *et al.*, 2006). Under these conditions, the risk of damage to DNA, cells, and tissue by reactive oxygen species (ROS) may be greatly reduced, and dietary antioxidants may have limited benefit to somatic growth (Pérez-Rodríguez, 2009). In support of this notion, a recent dietary study on the fast growing Booroolong frog (*Litoria booroolongensis*) found that dietary β-carotene supplementation significantly increased larval growth rate (Keogh *et al.*, 2018). Investigating differences in metabolic activity and ROS production between fast and slow growing frog species, and testing the relative importance of dietary carotenoids for somatic growth, could provide important insights into the physiological conditions under which these micronutrients are most beneficial.

An alternative explanation for the lack of growth effects is that carotenoids were preferentially invested into carotenoid-mediated traits that have a greater impact on fitness, driving a developmental trade-off among traits (Catoni *et al.*, 2008; Lin *et al.*, 2010). Dietary carotenoids are known to improve the coloration of *P. corroboree* (Umbers *et al.*, 2016), and a recent clay model experiment suggests that color functions as an aposematic signal (Umbers and Byrne unpublished data), so investment in colouration over growth may be strongly favored. Carotenoids may also be preferentially invested into immune function. While the relationship between immune function and carotenoid supplementation remains largely unexplored in amphibians (Cothran *et al.*, 2015; Szuroczki *et al.*, 2016), among other vertebrates carotenoid supplementation has been shown to improve antibody response and immunocompetence (Chew and Park, 2004; Duriancik *et al.*, 2010), which may take precedence over faster growth. Another possibility is that carotenoids are preferentially invested into protection from UV damage. *P. corroboree* inhabit high altitude regions that receive high levels of UV radiation (Colefax, 1956; Osborne, 1991), which is known to trigger ROS generation in amphibian skin cells (Stahl and Sies, 2002; Ogilvy and Preziosi, 2012). By acting as antioxidants, dietary carotenoids may play a photoprotective role in reducing UV damage to skin, limiting the availability of antioxidants for growth (Stahl and Sies, 2002; Ogilvy and Preziosi, 2012). As a first step towards understanding if there are developmental trade-offs among carotenoid-mediated traits in *P. corroboree*, it would be valuable to raise larvae under a broader range of carotenoid doses and determine how changes in carotenoid availability influence growth, colouration, immunocompetence, and UV damage.

Our finding that high doses of β-carotene had a significant effect on development, independent of any growth effects, has several possible explanations. First, β-carotene may be an important antioxidant during metamorphic climax, a process known to require significant energy expenditure, and to generate high levels of ROS (Inoue *et al.*, 2004; Stahl and Sies, 2005). By acting as an antioxidant, β-carotene may quench ROS and reduce them to a more stable state, limiting damage to DNA, cells, and tissue, which may allow for a faster transition through metamorphosis (Miller *et al.*, 1996; Stahl and Sies, 2003, 2005; Menon and Rozman, 2007). Our argument that β-carotene acts as an antioxidant against ROS during periods of extreme energy expenditure and metabolic activity in *P. corroboree* is supported by an earlier dietary study in this species which found that a broad-spectrum carotenoid supplement significantly improved exercise performance during escape-response episodes (Silla *et al.*, 2016). It is important to note, however, that the relationship between ROS production and metabolic activity is unlikely to be straightforward (Salin *et al.*, 2015). As such, further research is needed to quantify ROS production during larval development and metamorphosis in *P. corroboree* and determine the relationship between dietary β-carotene intake and circulating ROS levels. While this was not possible in the present study due to the critically endangered status of *P. corroboree* restricting opportunities for blood collection, such assays could be achieved in closely related species such as the common brown toadlet (*P. bibronii*).

A second way that β-carotene might have assisted development in *P. corroboree* is by activating genes that directly control development (Balmer and Blomhoff, 2002, 2006). Carotenes (like β-carotene) are precursors for vitamin A, and its derivative retinoic acid (Blomhoff and Blomhoff, 2006). Retinoic acid influences gene expression and protein production by activating pleiotropic genes which control the development of limbs, lung tissue, and the central nervous system (Balmer and Blomhoff, 2002, 2006). For anuran amphibians, metamorphosis involves a complete change in limb morphology, from tail loss to growth of quadrupedal limbs (Gosner, 1960; Wells, 2007). Furthermore, individuals transition from an aquatic to a terrestrial lifestyle, which requires the loss of gills and the growth of air-breathing lungs (Gosner, 1960; Wells, 2007). The addition of β-carotene to *P. corroboree* diets may have accelerated systemic morphological and physiological changes, resulting in a faster transition through metamorphic climax. To explore this possibility, it would be valuable to investigate the influence of β-carotene on gene expression during metamorphosis. This could be achieved using transcriptomic approaches such as DNA microarrays and next generation sequencing technologies (e.g. RNA-Seq), which are now being routinely used to understand the response of amphibians to various developmental environments (Jiang *et al.*, 2017).

Our finding that β-carotene, but not lutein, influenced development in *P. corroboree* supports our prediction that carotenoid classes will differ in their effects on developmental traits (Miller *et al.*, 1996; Stahl and Sies, 2003). The arrangement and number of conjugated double bonds, and the subsequent polarity of carotenoid compounds, are known to influence their antioxidant capacity (Miller *et al.*, 1996; Stahl and Sies, 2003). β-carotene may have had a positive effect on *P. corroboree* development due to its low polarity (resulting from its arrangement of conjugated double bonds), which increases its antioxidant capacity (Miller *et al.*, 1996; Stahl and Sies, 2003). In comparison, lutein may not have impacted development due to its high polarity (resulting from the presence of an alcohol group), making it less effective as an antioxidant (Miller *et al.*, 1996; Stahl and Sies, 2003). Notably, our finding that lutein did not have any effects on growth and development adds to a growing number of studies in anuran amphibians and other vertebrates reporting no effect of xanthophylls (predominantly lutein and zeaxanthin) on growth rate and/or developmental timing (Larcombe *et al.*, 2010; Butler and McGraw, 2012; Orledge *et al.*, 2012; Sutherland *et al.*, 2012; McCartney *et al.*, 2014; Cothran *et al.*, 2015). It may be the case that benefits of lutein manifest as improvements to general health and viability during adult life. For instance, lutein has been shown to have anti-inflammatory properties (Koutsos *et al.*, 2006), and also has the ability to increase bone density and mineralization (Takeda *et al.*, 2017). Considering this, it would be valuable for future lutein supplementation studies to measure the inflammatory response of individuals following an immune challenge, and/or x-ray individuals to quantify bone mass at metamorphosis (and in later life) to ascertain the effects of lutein on traits related to general health and viability.

Our finding that high (but not low) doses of β-carotene influenced development supports our prediction that the effects of carotenoids should be dose dependent. Low doses of β-carotene may not have influenced growth and development because available carotenoids were preferentially invested into fitness-determining traits that more directly impact survival, resulting in a developmental trade-off among traits (as discussed above). Alternatively, the low dose we used may have been too low to have any detectable effects on development. Notably, our findings are similar to those of a recent study in the Booroolong frog (*Litoria booroolongensis*), where 0.1 mg g^−1^ of β-carotene had no effect on growth and development, but 1 mg g^−1^ of β-carotene had a positive effect on both traits (Keogh *et al.*, 2018). Interestingly, this study also supplemented frogs with 10 mg g^−1^ of β-carotene, and, at this relatively high dose, found a negative effect on growth and development. Based on this finding we might also expect detrimental effects in *P. corroboree* at doses beyond 1 mg g^−1^. An important area for future research will be identifying the thresholds at which carotenoids have beneficial effects, both during development and post-metamorphosis. More broadly, identifying optimal supplementation doses in *P. corroboree* and other frog species will be an essential step towards understanding broad taxonomic patterns in carotenoid dose thresholds in anuran amphibians.

Our finding that a high dose (1 mg g^−1^) of β-carotene can accelerate development has important conservation implications because *P. corroboree* is listed as critically endangered and is the subject of a long-term conservation breeding program (Hunter *et al.*, 2007). The capacity to more rapidly generate metamorphs is likely to benefit this program by allowing managers to more effectively replenish captive colonies with new cohorts, and avoid problems associated with natural attrition and/or inbreeding. Of equal importance, the more rapid generation of metamorphs could benefit the reintroduction of *P. corroboree* in various ways. First, generating metamorphs more rapidly could increase the number of individuals available for release, which may improve reintroduction success if there are positive correlations between population density and individual fitness (i.e. the allee effect) (Armstrong and Seddon, 2008). Moreover, individuals who metamorphose faster could be maintained on adult diets for longer periods prior to release, which would provide an opportunity to release larger animals. This is critical because larger body size can significantly reduce an individual’s risk of mortality resulting from starvation, predation, competition, and desiccation (Cabrera-Guzmán *et al.*, 2013). In addition, a recent study revealed that smaller anurans carry higher infection loads of chytrid fungus (responsible for the fatal skin disease chytridiomycosis) and bear higher energetic and osmoregulatory costs, exposing them to a higher risk of mortality resulting from disease (Wu *et al.*, 2018). In anuran amphibians, body size at metamorphosis is also a strong predictor of reproductive success in both sexes (Wells, 2007, Amézquita A, and Lüddecke H, 1999). As such, larger body size at release may improve the lifetime fitness of reintroduced frogs. Finally, although we found no evidence for a beneficial effect of lutein on growth and development, we also found no evidence for detrimental effects. Given that lutein may be beneficial for general health and viability of individuals, there may be value in including this micronutrient in captive diets. More broadly, because captive breeding is a standard recovery action for threatened anuran amphibian species globally, dietary supplementation of β-carotene (and potentially other carotenoids) may have widespread conservation value. As such, we recommended ongoing experimental tests of the effects of dietary carotenoids on growth and development (and other fitness-determining traits) across a diversity of threatened amphibian species.

## Supplementary Material

Supplementary Data
