# Supplementary material for: Effect of carotenoid class and dose on the larval growth and development of the critically endangered southern corroboree frog
Source: Conserv Physiol. 2019 Mar 13;7(1):coz009. doi: 10.1093/conphys/coz009 (PMC6425257; doi:10.1093/conphys/coz009)
Supplement: Supplementary Data [file coz009_mcinerney_et_al_cons_physiol_supplementarymaterial_feb2019.docx]

**Supplementary Material**

**Effects of carotenoid class and dose on the growth and development of the critically endangered southern corroboree frog (*Pseudophryne corroboree*)**

**Emma P. McInerney^1^*, Aimee J. Silla^1^, Phillip G. Byrne^1^**

^1^School of Earth, Atmospheric and Life Sciences, University of Wollongong, NSW 2522, Australia

Corresponding author: pbyrne@uow.edu.au

Ph: +61 2 4298 1932

**Supplementary Table S1.** Composition of carotenoid diets fed to larval *P. corroboree*.

| Diet treatment | Carotenoid class | Carotenoid type | Carotenoid mass (g) | Cellulose mass (g) | Fish flake mass (g) | Total feed mass (g) |
| --- | --- | --- | --- | --- | --- | --- |
| Control | - | - | 0.000 | 0.001 | 1.000 | 1.001 |
| Low-dose β-carotene | Carotene | β-carotene | 0.0001 | 0.0009 | 1.000 | 1.001 |
| High-dose β-carotene | Carotene | β-carotene | 0.001 | 0.000 | 1.000 | 1.001 |
| Low-dose lutein | Xanthophyll | Lutein | 0.0001 | 0.0009 | 1.000 | 1.001 |
| High-dose lutein | Xanthophyll | Lutein | 0.001 | 0.000 | 1.000 | 1.001 |

**Supplementary Table S2.** Composition of the control diet fed to larval *P. corroboree*

| Component | Unit | Mass |
| --- | --- | --- |
| Protein | (g/100g) | 44.400 |
| Carbohydrate | (g/100g) | 28.000 |
| Ash | (g/100g) | 10.900 |
| Fat | (g/100g) | 8.500 |
| Moisture | (g/100g) | 8.000 |
| Vitamin E | (mg/kg) | 78.700 |
| Total Carotenoids | (mg/kg) | 14.540 |
| -β-carotene | (mg/kg) | 5.466 |
| -Lutein | (mg/kg) | 2.268 |
| -Astaxanthin | (mg/kg) | 0.233 |
| Vitamin A | (mg/kg) | 1.880 |

Shown are the results of proximate analysis conducted by the Australian National Measurement Institute using the following analytical standards: AOAC 18^th^ edition 2005; moisture (950.46), fat (948.15), protein (981.10), and ash (923.03). In addition to analysis of vitamin A, vitamin E and carotenoids by the Animal Health and Research Centre at Adelaide Zoo using reverse phase high performance liquid chromatography (HPLC).
